# Superinvolution of the Uterus Following Trachelorrhaphy

**Published:** 1888-11

**Authors:** Virgil O. Hardon

**Affiliations:** Professor of Obstetrics and Diseases of Women and Children, Atlanta Medical College, Atlanta, Ga.


### Selections.

### SUPERINVOLUTION OF THE UTERUS FOLLOWING TRACHELORRHAPHY.*

BY VIRGIL O. HARDON, M. D.,

Professor of Obstetrics and Diseases of Women and Children, Atlanta Medical College, Atlanta, Ga.

In August, 1887, I reported, with brief comments, two cases of what I termed "superinvolution of the uterus following trachelorrhaphy."† In that report I expressed the conviction, founded upon an examination of the literature at my disposal, that no similar cases had previously been published. Further investigation has proven the correctness of this opinion. By correspondence with a large number of the leading gynecologists of this country and of Europe, I have, however, been able to collect seven hitherto unpublished cases, thus showing that my experience has not been unique, and demonstrating the possibility of the occurrence of superinvolution as a troublesome sequel of Emmet's operation for the repair of the lacerated cervix. I propose in this paper to consider the pathology of this condition and to report the cases which I have been able to gather.

Involution is the term applied to the physiological process by which the womb returns to its non-puerperal condition after labor. The most generally accepted theory in regard to the manner in which this change is effected is that advanced by Heschl.‡ This theory is summarized by Lusk as follows: "The processes are inaugurated at the commencement of labor. During the rapidly

*From American Journal of Obstetrics,

†Atlanta Medical and Surgical Journal, August, 1887.

‡Zeitschr, d. K. K. Gesellsch, d. Aerzte zu Wien, VIII Jahrg., zweite Halfte, 1852, S. 228 u. ff.

following contractions of the uterus, the cell elements are consumed, while at the same time the compression of the nutrient vessels cuts off fresh supplies from the oxidized protoplasm. The fatty degeneration of the muscular fibres continues after the expulsion of the ovum. The contractions which bear the name of after-pains point to the continuance of muscular cells capable for a time of functional performance. Gradually, however, the proteine substances are converted into fats which undergo absorption. Whether the enormously enlarged cells of pregnancy ever entirely disappear is still an open question. In the fourth week, young cells of new formation make their appearance upon the external layer of the uterus, from which eventually a new uterus is developed. Thus destruction and reparation go hand-in-hand. In from six to eight weeks, the process described reaches its completion."*

Subinvolution is defined, in one of the most recent works on gynecology, as a prolongation or arrest of the process of involution†. This definition fails, however, to express in its entirety the actual condition under such circumstances. It is true that if during the process of involution the circulation of blood in the uterus is deranged by any irritating cause, a congestion of the organ follows, and the muscular fibres receive sufficient blood to furnish them the material for the continuance of a certain amount of their vital activity, so that, instead of undergoing fatty degeneration, they continue for a time to enjoy a low grade of existence. But what is of greater importance than this partial persistence of the hypertrophied muscular fibres is the fact that an active proliferation of connective tissue ensues. After a variable length of time, this proliferation reaches its limit, the newly formed connective tissue undergoes contraction, and the uterine walls present more or less of a dense, white, cartilaginous appearance, and sometimes even creak under the knife when cut. At this stage the connective tissue in the womb may exceed the muscular tissue in quantity.‡

*Lusk, "Science and Art of Midwifery," 2d ed., p. 241.

†Byford, "Diseases of Women," 4th ed., p. 563.

‡Klob, "Pathol. Anat. d. Weibl. Sexualorg.," Wien, 1864.

Subinvolution of the uterus involves, therefore, three factors: 1st, congestion of the organ; 2d, failure of the muscular structure to undergo complete degeneration and replacement; 3d, excessive development of the intermuscular connective tissue. The microscopical researches of Beck,* Klob,† and Sinety‡ agree in showing that the excessive development of connective tissue is the essential pathological condition. Hyperemia is recognized as a prominent feature in the early stage of subinvolution, as is shown by the fact that it is the condition to which treatment is always directed during this stage. Thomas says: "The element which sustains the disease is an excessive supply of blood."§ But as the morbid process develops, this factor gradually diminishes, and as contraction of the organ progresses, sometimes after months and sometimes after years have elapsed, it may disappear altogether, and a condition be reached in which the womb is scarcely more than a ball of hard fibrous tissue, poorly supplied with blood, and retaining only a small portion of its original muscular structure. This is the condition which has been variously termed "chronic metritis,"|| "diffuse connective-tissue proliferation,"¶ "areolar hyperplasia,"** and "sclerosis uteri."††

The most common cause of subinvolution of the uterus is conceded to be laceration of the cervix. Emmet says: "For many years past I have met with few or no cases of subinvolution which were not due to laceration of the cervix."‡‡ When a laceration has occurred, nature sets about repairing the injury by the usual procesess of inflammation and granulation—processes which are antagonistic to the processes of degeneration and involution which are going on in the womb at the same time. The consequence is that neither process is perfectly formed. The wound in the cervix is not readily healed, but remains for a time

*London Obstet. Trans., vol. xiii., p. 239. †Op. cit. ‡"Gynecologie," Paris, 1879, pp. 315, 351.

§"Diseases of Women," New York, 1880, p. 317.

||Scanzoni, "Die Chronische Metritis," Wien, 1863.

¶Klob, op. cit.

**Thomas, op. cit. ††Skene, Am. Jour. of Obst., vol. V., pp. 387, 481.

‡‡"Principles and Practice of Gynecology," 3d ed., p. 439.

as a granulating surface, causing the womb to become permanently congested under the continuous efforts of nature to accomplish the reparative process. The overweighted womb sinks below its normal position in the pelvis, its circulation is thereby interfered with, and a venous stasis results, increasing and perpetuating the already existing congestion. This continued congestion forms the starting point of the condition of subinvolution after the manner already described.

Since the womb, when unaided, has no tendency to rid itself of this congestion, the first stage of subinvolution, the stage of hyperemia, is usually one of long duration, so long, indeed, that in the majority of cases, unless arrested by treatment, it persists until the occurrence of the physiological menopause. The symptoms of this stage are such as result from increased weight of the uterus and reflex manifestations of irritation of the uterine nerves. In exceptional cases, however, the hyperemia disappears before the occurrence of the menopause, thus inducing the second stage of subinvolution, the stage of so-called "sclerosis uteri," whose symptoms consist only of the nervous reflexes and scanty menstruation or absolute amenorrhea. In some cases the nervous reflexes are absent, and scanty menstruation or entire amenorrhea constitutes the only symptom. The condition thus reached is the same as that which has been described by various authors under the name of superinvolution.

Sir Jas. Y. Simpson thus describes a case of superinvolution following labor, in which he was so fortunate as to obtain an autopsy: "The uterus was very small and atrophied in its length and breadth, its size being diminished about a third below the natural standard in all its measurements, and its parietes were correspondingly thin and reduced. The whole length of the uterine cavity, from the os to the fundus, was not more than one inch and a half. The tissue of the uterus appeared dense and fibrous."* Thomas says, in speaking of the final stage of subinvolution: "Every practitioner must have met with cases in which a large, red, engorged and soft uterus, examined after an interval of several years, has been found, to his surprise, to

*"Diseases of Women," New York, 1872, p.98 5.

have become small, densely hard, white and anemic, and its cavity diminished in size. Such an organ removed from the body cuts like fibrous tissue, and appears when cut almost as dense and bloodless."* The identity of the two conditions is obvious.

Thus it would appear that superinvolution is simply the final stage of subinvolution, or, as Reamy states it, "the tendency of this disease (subinvolution), untreated and uncomplicated, is to the establishment of chronic metritis and later sclerosis of the uterus, the so-called superinvolution." †

It is evident that, if by artificial means the hyperemia, which forms a distinguishing feature of the first stage of subinvolution, be made to disappear, the resulting condition may be, to a greater or less degree, the same as when the second stage is reached in the uninterrupted course of the disease. It has been shown that, in subinvolution from laceration, this hyperemia is perpetuated by the mechanical injury to the womb and the downward displacement of the organ. Clinical experience has domonstrated that when this injury is repaired by trachelorrhaphy, the hyperemia disappears and the womb gradually returns to its normal condition. This is the usual result of trachelorrhaphy. But if, previous to the operation, the connective-tissue proliferation has progressed to such a degree that nature is unable to accomplish a *restitutio ad integrum*, after the exciting cause is removed, the operation will not have cured the subinvolution, but, by removing the hyperemia, will simply have precipitated the final stage of the disease, the stage of sclerosis uteri or superinvolution. It is thus that the occasional occurrence of superinvolution, as a sequel of trachelorrhaphy, appears to me to admit of rational explanation.

In a recent article by Saenger, of Leipsic, upon "The Involution of the Muscular Tissue of the Puerperal Uterus,"‡ the views of Heschl of involution by fatty degeneration and replacement are combated, and a new theory is advanced, based upon

*Op. cit., p. 320.

†"Amer. System of Gynecology," vol. I., p. 659.

‡"Annals of Gynecology," July, 1888.

microscopical examination of seventeen uteri. Two of these were normal, unimpregnated organs of women who had borne children; two at the sixth and eighth months respectively of gestation; twelve puerperal, representing stages of the puerperium varying from four hours to fifty-five days after labor; while the seventeenth specimen was one in which pregnancy had been present in the rudimentary horn of a *uterus bicornis*. This article bears so directly upon the subject under consideration that I feel warranted in quoting certain of Saenger's conclusions in full. His fifth, seventh, ninth and tenth propositions are as follows:

(5.) Probably not a single muscular fibre is destroyed by complete fatty degeneration. The regressive changes within the puerperal muscular fibres, which may be denominated *paratrophic*, have for their object only the true involution of the muscular fibres until they have attained their earlier size and form. The definition of atrophy as a pathological process does not correspond with the physiological nature of these processes.

(7.) The intermuscular connective tissue experiences a similar involution in its cellular and fibrillar elements; thus it does not play any active part nor experience hypertrophy.

(9.) *Post-partum* subinvolution of the uterus is not an independent disease, but a prolonged and incomplete involution, which is disturbed in its progress, and is dependent upon changes in position, disturbances in the circulation and inflammations of the uterus and its surroundings.

(10.) By *post-partum* atrophy of the uterus is signified a diminution in its size which brings it to the boundary line of the pathological, so that its volume may sink manifestly below that of the normal pluriparous uterus, this being conditioned upon an abnormally great reduction in the muscular fibres and the intermuscular connective tissue, with an anemia of the uterus, the sexual organs in general, or the entire body as a probable fundamental cause. In physiological cases the nutrition improves, the increase in the volume of the uterus is continued until the normal is again reached and menstruation returns as a regular and uninterrupted function. In pathological cases the uterus remains permanently atrophic with the continuance of oligomenorrhea or amenorrhea.

If these views shall be corroborated by other competent observers, our prevailing theories in regard to subinvolution and superinvolution must undergo decided modification. From Saenger's standpoint it will be very difficult to explain those cases of sclerosis uteri following subinvolution in which there is not only relative, but absolute hypertrophy of connective tissue, such as have been observed by competent authorities too frequently to admit of any doubt of their existence.

The cases of superinvolution which I have reported find their explanation, in Saenger's opinion, in his tenth proposition quoted above. He regards them simply as cases of normal *post-partum* atrophy, following the completion of involution after the operation of trachelorraphy. In a private communication which I received from him he states that such is his view, and expresses the opinion that without any treatment my two patients would have resumed their normal menstrual functions in the natural course of events. Granting his theory of involution to be correct, it appears to me more rational to place my cases in the category referred to by him of "pathological cases in which the uterus remains permanently atrophic with the continuance of oligomenorrhea or amenorrhea." In this connection it is interesting to note the fact that, of the nine cases collected by me and reported herewith, in eight, which were treated by intrauterine faradization, the menses returned, while in one, which had no treatment, the menses did not return (Case III).

In response to letters of inquiry sent to leading gynecologists of this country and of Europe, fifty-six replies have been received. Of this number, fifty-three state that no cases similar to those reported by me had been observed by the writers. From the remaining three replies, reports of seven cases were obtained: one from Dr. A. Reeves Jackson, of Chicago; two from Dr. Geo. F. Hulbert, of St. Louis, and four from Dr. C. Kollock, of Cheraw, S. C. Through the courtesy of these gentlemen I am permitted to present herewith their reports of these cases, together with my own two.

Page(s) missing

CASE I.—Mrs. H. B. L., white, married, aged 32, suffered laceration of the cervix in her third labor six years ago. Since that time she has experienced bearing-down sensations, backache, pain in loins, frequent micturition, leucorrhea, menorrhagia, headache, insomnia, neuralgia, mental depression and indigestion. Examination showed a bilateral laceration of the cervix, the womb in a state of subinvolution, three inches deep. June 7th, 1886, trachelorrhaphy was performed, perfect union followed, and patient returned to her home. Three and half months later she was seen by me, and was then free from all her former symptoms, both local and general.

In March, 1887, nine months after the operation, the menses, which had been normal since the operation, began to become scanty. At the same time there developed neuralgia of the head and back of the neck, with pains up and down the spine. Vertigo, dimness of vision so that she was unable to read or write, insomnia, great mental depression with hysterical tendencies, loss of appetite and flesh, all followed, so that by mid-summer she seemed a complete wreck.

On the 16th of August, she entered my private infirmary for treatment. Examination showed the laceration to have been completely and permanently cured. The womb was small, being scarcely two inches deep, and felt dense and hard to the touch. The cervix was small, and the canal with difficulty admitted a Simpson's uterine sound. No tenderness or hardness in the cellular tissue, no leucorrhea and no local pain. The menses lasted only a few hours at each period and were pale and watery.

The patient was placed upon nourishing diet and a tonic of iron, quinine and strychnia. The faradic current of electricity was applied directly to the womb for half an hour on every second day. The positive pole was placed in the uterine cavity and the negative pole applied to the hypogastrium. This treatment was continued for three months. Under the stimulus of the electric current the womb gradually increased in size until it reached a depth of 2½ inches. The menses were restored to their normal color, quantity and duration. The nervous symptoms gradually disappeared and the patient recovered her full strength and health.

CASE II.—Mrs. J. T. S., white, aged 27. Always well until the birth of her child two years ago. Since then has suffered from backache, pain in the pelvis, difficult and frequent micturition, painful defecation, nervous depression, insomnia and indigestion. The indigestion has become a very prominent feature, and during the past year she has been under constant treatment for this symptom by various physicians without relief. Examination showed the womb to be enlarged, 3¼ inches deep, low in the pelvis and tender to the touch. Laceration of the cervix on the left side, with eversion of the lips. February 26th, 1887, trachelorrhaphy was performed and perfect union followed. The relief of the symptoms was immediate. The persistent indigestion which had resisted all remedies was at once relieved and did not return. The local pains disappeared and the nervous symptoms subsided. The two menstrual periods following the operation were normal in all respects. In May the flow was very scanty, lasting only a day and a half, and in June did not appear at all. In all other respects the patient's health remained good. Examination made July 1st showed the womb to be only two inches in depth and hard and dense to the touch. The laceration was entirely cured, and there was no pain or hardness in any portion of the pelvic cellular tissue. Intra-uterine faradization was applied three times a week and daily for the week preceding the time of the menstrual period. The menses appeared for two days in July and three days in August. The electrical treatment was continued for two months, during which time the womb gradually increased to a depth of 2⅜ inches, and the menses became normal and remained so until the patient became pregnant in October, 1887. She was delivered by me in July following is now in perfect health.

CASE III.—(By Dr. A. Reeves Jackson.) "Mrs. A. W., 34 years of age, was delivered of her fourth child by the aid of forceps. She left her bed at the end of three weeks, but soon found that she was not so capable as she had been on former similar occasions. On examination, at the end of two months after confinement, her physician discovered a bilateral laceration of the cervix with marked eversion. A bloody discharge had continued since the birth of the child. Two months later I saw her. A bloody mucus in great abundance was issuing from the cervical canal and from the exposed cervical endometrium. The uterus was soft, tender, heavy, and the depth of the canal, measuring from the proper uterine mouth, was nearly four inches.

"Trachelorrhaphy was performed September 9th, 1885, four months after delivery. Recovery was satisfactory. In the following February her physician informed me that a menstrual period had appeared in November, without pain, the amount of discharge being decidedly less than the patient had ever previously known. In August, 1886, the latter came to the city for the purpose of having me determine the possible presence of pregnancy. After the scanty flow in November, she had menstruated with entire regularity for five months, the quantity of discharge becoming each time less, until at the last, which occurred in April, a mere light-reddish stain was apparent on the napkin. From that time onward she had adopted the theory of pregnancy, although all other usual symptoms of that condition were absent.

"On examination, I found the uterus atrophied in every dimension. It seemed about two inches in length, as defined by bimanual, but the os externum was closed, and, therefore, I could not ascertain the depth of the canal. Her health was apparently perfect and no treatment was advised. There had been no moliminal symptoms for the past few months. The uterus and ovaries had apparently gone out of business together."

Dr. Geo. F. Hulbert writes: "I have met this condition in only two cases out of one hundred operated upon by me."

CASE IV.—"In one of these there was nothing to denote any trouble save an absolute cessation of menstruation. This case came back to me for treatment one year after the operation under the impression that I had closed up the mouth of the womb so completely that the blood could not get out.

"Close questioning established the fact that there had not been the slightest menstrual molimen during the time. Patient was, to all intents and purposes in a normal condition, save the delusion afore-mentioned. The uterus was infantile in size, one and a half inches in depth. There were no evidences of any diseased condition about the adnexa. The ovaries could be felt, and they, too, had become much smaller than normal. Electricity in two months relieved the condition, and a year after I delivered her of her second child."

CASE V.—"The other case presented locally like features to the above, save the ovaries were not involved—at least to the same degree—for the size was not so small and they possessed a degree of sensitiveness more nearly approaching the normal. Menstrual molimina were present in this case, and during this time the sensitiveness was more pronounced. This patient was a victim to melancholia and neuralgia of the fifth nerve, which made her existence miserable. There was a history of neurotic troubles in her family, an uncle having been insane. In this case electricity accomplished a return of the menstrual function in six weeks. This patient about a year after returned to me again suffering from amenorrhea for a period of three months. Examination revealed pregnancy.

"In the first case (Case IV.) menstruation ceased after the second appearance following the operation. In the second (Case V.) it was after the fifth month. In both the operation was performed a few months (two to three) after the parturition that caused the laceration and before the menses had become established. In both, however, menstruation came on after the operation, but only temporarily."

CASE VI.—(By Dr. C. Kollock, of Cheraw, S. C.) "Mrs. I. T. G., aged 31, has had five children, never had an abortion; a complete blonde, light hair, blue eyes, fair and very clear complexion; was brought up in affluence, never knew anything of hardship. General health always good, is very intellectual and highly cultivated, nervous and excitable, though generally has good control of herself. Has never had the slightest trouble in menstruation until within the last two years. Youngest child is now three years of age. In her last confinement she had a bilateral laceration of the cervix uteri. Since that time she has suffered all the usual tortures of subinvolution of the uterus, and life was a burden until I did the operation of trachelorrhaphy on the 16th of January, 1887. She lives some distance from me, but remained with me nearly two months after the operation. The operation was a complete success, and all unpleasant symptoms, rational and physical, disappeared before she left for her home in North Carolina. I received letters from her repeatedly in which she always spoke of the great improvement in her health. In the latter part of November, 1887, I got a letter from her, from the tone of which I judged she was very miserable. I immediately wrote her to come at once to Cheraw, which she did about the 10th of December last. Upon examination I found her suffering from all the signs of superinvolution. The uterus was reduced almost to a state of senile atrophy. Depth scarcely two inches, cervix hard and small. Menstruation scanty, thin and watery, sometimes missing. In conjunction with tonic treatment, intra-uterine faradization was applied three or four times a week. In about two months, under this treatment, the womb increased to two and a half inches in depth, menstruation became normal, and the patent returned to her home in good health."

CASE VII.—(Dr. C. Kollock.) "A lady who lives some distance from Cheraw came to be treated by me about six weeks ago. She had trachelorrhaphy done by another physician in November, 1886. Was entirely relieved, she tells me, of all the unpleasant symptoms of subinvolution till about the 1st of October, 1887, when menstruation became irregular, was thin and colorless and very scant. When she came to me she had not menstruated in six weeks, was low spirited, had constant backache, neuralgia at times, and was much troubled with indigestion and insomnia. Examination showed the uterus in an atrophic condition, much resembling that which occurs after the menopause, and hardly measuring two inches in length. Under a general tonic treatment of iron, quinine and strychnine, and applications of electricity to the uterus three times a week, she now shows improvement. She menstruated about two weeks ago with decided improvement, both in quantity and quality."

CASES VIII. and IX.—(Dr. C. Kollock.) "Two other cases upon which I had operated for laceration of the cervix were in pretty much the same condition and presented the same symptoms. I put them on a general tonic treatment of iron, quinine, and strychnine and applied electricity to the uterus three times a week. These patients are beginning to show signs of improvement now, and I see no reason why they should not be entirely relieved in the course of three or four months."

